# Editorial: Hereditary Optic Neuropathies: A New Perspective

**DOI:** 10.3389/fneur.2021.742484

**Published:** 2021-09-22

**Authors:** Valerio Carelli, Rustum Karanjia, Chiara La Morgia

**Affiliations:** ^1^Istituto di Ricerca e Cura a Carattere Scientifico Istituto delle Scienze Neurologiche di Bologna, Programma di Neurogenetica, Bologna, Italy; ^2^Department of Biomedical and Neuromotor Sciences (DIBINEM), University of Bologna, Bologna, Italy; ^3^Doheny Eye Institute, Los Angeles, CA, United States; ^4^Department of Ophthalmology, Doheny Eye Center, David Geffen School of Medicine, University of California, Los Angeles, Los Angeles, CA, United States; ^5^Department of Ophthalmology, Ottawa Eye Institute, University of Ottawa, Ottawa, ON, Canada; ^6^Ottawa Hospital Research Institute, Ottawa, ON, Canada; ^7^Istituto di Ricerca e Cura a Carattere Scientifico Istituto delle Scienze Neurologiche di Bologna, UOC Clinica Neurologica, Bologna, Italy

**Keywords:** optic nerve, gene therapy, retinal ganglion cells, Leber's hereditary optic neuropathy (LHON), induced pluripotent stem cells (IPSCs)

Over 30 years have elapsed from the landmark finding of the first point mutation in mitochondrial DNA (mtDNA) associated with Leber's hereditary optic neuropathy (LHON) by Doug Wallace and his team in 1988 (1). Twelve years later came the identification of the nuclear *OPA1* gene, which encodes for a mitochondrial dynamin-like protein, whose pathogenic mutations cause Dominant Optic Atrophy (DOA) (2, 3). This was a predictable convergence, as both nuclear and mtDNA mutations may cause a similar mitochondrial disorder resulting in vision loss, identifying DOA as a mitochondrial disease similar to LHON (4, 5). We are delighted to have compiled this special issue of Frontiers in Neurology on hereditary optic neuropathies, to highlight how lively the field of inherited optic neuropathies is in 2021.

Three of the papers published in this series deal with new mtDNA variants affecting complex I in association with LHON, highlighting the emergence of the *MT-ND5* gene as a new hot spot for LHON mutations (Sun et al.; Engvall et al.; Peverelli et al.). Thus, quoting Salvatore DiMauro, these findings clearly indicate that “*we are not scraping the bottom of the barrel*” yet (6). Complex I remains the most frequent biochemical defect and LHON-like phenotypes may frequently overlap with more complex phenotypes combining MELAS and Leigh syndrome features in a continuum (7).

LHON remains a paradigm for mitochondrial disorders and neurodegeneration, thus continuous efforts are directed to the intimate understanding of its pathogenic mechanism. The *in vitro* modeling of disease has become key to preclinical science, and the use of reprogrammed pluripotent stem cells (iPSCs) derived from primary LHON patient's cells, into differentiated neuronal cellular types including retinal ganglion cells (RGCs) is expanding our toolbox to understand and treat this and other diseases (Peron et al.). This emergent biotechnology to model rare disorders such as LHON, also overcomes some of the difficulties of using animal models, which do not always faithfully reproduce mitochondrial diseases. The innovative models like iPSCs-derived organoids have helped to untangling the details of the pathogenic mechanisms allowing multiomic approaches at single cell type level (8), truly applying the principles of personalized medicine, as summarized in a perspective article in this special issue (Peron et al.).

RGCs are special neurons that are functionally asymmetric and metabolically skewed by the architecture of their axons (4, 5). The need to keep the inner retina transparent to light requires the RGC axons to remain unmyelinated for their long intra-retinal segment, before converging at the optic disc to form the myelinated optic nerve. Understanding this special neuronal cell type is essential to dissect hereditary optic neuropathies and this aspect is comprehensively covered in a complete review of the 18 RGCs subtypes and their morphological and functional characteristics as part of this special issue (Kim et al.). This review also looks at the tools to explore RGCs and how they are eventually lost in different pathological conditions including inherited and acquired optic neuropathies.

DOA, as previously stated, is a companion disease to LHON, which in the large majority of cases is due to various types of pathogenic mutations in the *OPA1* gene (2, 3). This gene encodes a protein that comes in 8 isoforms further processed from long to short forms, providing a “kaleidoscopic” spectrum of functions, a concept which is comprehensively reviewed in this special issue, covering the wide range of models used to study OPA1 to date (Del Dotto and Carelli) The complexities of OPA1 function and dysfunction may include mechanisms regulating the cellular resilience during development and adulthood, such as adaptations to OPA1 deficiency that may be reflected in mitochondrial motility or inflammatory responses, which ultimately impinge on cell aging, as argued in a dedicated article in this special issue (Erchova et al.).

Understanding the clinical presentation of these diseases continues to evolve and the use of new objective clinical metrics, such as the photopic negative response (PhNR), are becoming more important as new treatments are being developed (Botelho et al.). Again, LHON is on the frontline of new treatment strategies with gene therapy trials, which have been recently concluded, and the first and up to now the only therapy approved for a mitochondrial disorder by the European Medicines Agency, the quinone analog idebenone. These clinical topics have been reviewed in a contribution to this special issue (Hage andVignal-Clermont). Moreover, a comparison between the recently published results of gene therapy trials and the natural history of LHON is included in this special issue and provides an interesting perspective on the success of these treatments (Newman et al.).

Overall, the field of hereditary optic neuropathies remains extremely dynamic and lively. By presenting the “*tip of the neurodegeneration iceberg*” in this special issue we hope to demonstrate the importance of these diseases as an informative model to make key observation of relevance to other neurodegenerative disorders (9). From the genetic foundation, to the clinical and preclinical research, and ultimately ending with new therapeutic strategies, we look forward to further groundbreaking progress in hereditary optic neuropathies, leading to the ultimate goal of an effective cure for these patients, preserving vision.

## Author Contributions

All authors listed have made a substantial, direct and intellectual contribution to the work, and approved it for publication.

## Funding

Research on hereditary optic neuropathies, over the last ten years, has been supported by the Italian Ministries of Health and of Research (to VC and CLM); Telethon-Italy (Grant numbers GGP06233, GGP10005, GGP11182, and GGP14187) (to VC); Programma di ricerca Regione-Universit. 2010-2012 (Grant number PRUa1RI-2012-008) (to VC); and the patient-led organisations (IFOND, UMDF, MITOCON, The Poincenot Family, and the Gino Galletti Foundation) and patient's donations (to VC). RK or his institutions have received funding from the following organizations: 1. Stealth BioTechnology, Boston, MA, United States. 2. PTC Therapeutics, South Plainfield, New Jersey, United States. 3. GenSight Biologics S.A. Paris, France. 4. International Foundation for Optic Nerve Disease, New York, United States. 5. United Mitochondrial Disease Foundation, Pittsburgh, Pennsylvania, United States. 6. National Aeronautics and Space Administration, Washington, D.C., United States.

## Conflict of Interest

VC is as scientific consultant in boards of GenSight Biologics, Stealth BioTherapeutics, Santhera Pharmaceuticals and Chiesi Farmaceutici. VC received speaker honoraria from Chiesi Farmaceutici and an unrestricted research grant from Stealth BioTherapeutics. CLM received speaker's honoraria and travel support from Santhera Pharmaceuticals and consulting fees from Regulatory Pharmanet and Chiesi Farmaceutici. RK received consulting and honoraria fees from Stealth BioTherapeutics and Chiesi Farmaceutici.

## Publisher's Note

All claims expressed in this article are solely those of the authors and do not necessarily represent those of their affiliated organizations, or those of the publisher, the editors and the reviewers. Any product that may be evaluated in this article, or claim that may be made by its manufacturer, is not guaranteed or endorsed by the publisher.
